# PEDF inhibits LPS-induced acute lung injury in rats and promotes lung epithelial cell survival by upregulating PPAR-γ

**DOI:** 10.1186/s12890-023-02666-3

**Published:** 2023-09-23

**Authors:** Lei Xu, Yifei Chen, Shoujie Feng, Zeyan Liu, Ying Ye, Ranran Zhou, Lijun Liu

**Affiliations:** 1 Department of Emergency Medicine, The Second Affiliated Hospital of Soochow University, Suzhou, 215004 Jiangsu China; 2 Department of Emergency Medicine, The Affiliated Hospital of Xuzhou Medical University, Xuzhou, 221002 Jiangsu China; 3 Department of Emergency Medicine, The Affiliated Hospital of Yangzhou University, Yangzhou, 225001 Jiangsu China; 4 Thoracic Surgery Laboratory, Xuzhou Medical University, Xuzhou, China; 5 Department of Thoracic Surgery, The Affiliated Hospital of Xuzhou Medical University, Xuzhou, 221002 China; 6 Department of Emergency Medicine, The Second Affiliated Hospital of Anhui Medical University, Hefei, 230601 Anhui China

**Keywords:** PEDF, ALI, Anti-inflammatory, Epithelial cell, PPAR-γ

## Abstract

**Background:**

The progression of acute lung injury (ALI) involves numerous pathological factors and complex mechanisms, and cause the destruction of epithelial and endothelial barriers. Pigment epithelium-derived factor (PEDF) is an angiogenesis inhibitor and a potential anti-inflammatory factor. The purpose of this study was to investigate the effect of PEDF on lipopolysaccharide (LPS)-induced ALI in rats.

**Methods:**

In vivo, pathological and injury related factors examination were performed on rat lung to investigate the effect of PEDF on ALI. In vitro, the effect of PEDF on inflammatory injury and apoptosis of lung epithelial type II RLE-6TN cell was evaluated, and the expression of inflammatory factors and related pathway proteins and PPAR-γ (in the presence or absence of PPAR-γ inhibitors) were analyzed.

**Results:**

In vivo results showed that PEDF inhibited the inflammatory factor expression (TNF-α, IL-6 and IL-1β) and progression of ALI and reduced lung cell apoptosis in rats. In vitro results showed that PEDF could effectively inhibit LPS-stimulated inflammatory damage and apoptosis of RLE-6TN cells. PEDF inhibited the RLE-6TN cell injury by enhancing the expression of PPAR-γ.

**Conclusions:**

PEDF is an anti-inflammatory factor, which can inhibit apoptosis of lung epithelial cells by upregulating the expression of PPAR-γ and reducing LPS-induced ALI in rats.

**Supplementary Information:**

The online version contains supplementary material available at 10.1186/s12890-023-02666-3.

## Introduction

Acute lung injury (ALI) and its accompanying complication, acute respiratory distress syndrome (ARDS), is a severe acute progressive hypoxic respiratory failure caused by numerous direct and indirect factors [1, 2]. Globally, the fatality rate of ALI/ARDS is high (27–45%), and patients account for approximately 10% of hospitalized intensive care unit patients [3]. Therefore, it is of great significance to develop safe and effective therapeutic drugs to treat the condition.

It is not yet clear what mechanism causes the rapid deterioration of ALI, but there is growing evidence that lung epithelial cell damage is often the initiating factor [4, 5]. Lung epithelial cells are an important line of defense against pathological stimuli. Diffuse alveolar injury and lung epithelial cell death are both prominent features of ALI [2]. The death of lung epithelial cells induces the release of damage-related molecular pattern molecules, which then go on to trigger and maintain inflammation, ultimately leading to an uncontrollable cascade of inflammation [6]. A complete alveolar epithelial barrier plays an important role in altering the prognosis of acute lung injury as well as the initial occurrence and development of the disease [7]. Therefore, maintaining the integrity of the lung epithelial barrier and reducing alveolar epithelial cell apoptosis are both key to the effective treatment of ALI/ARDS.

Pigment epithelium-derived factor (PEDF) is an endogenous protein composed of 418 amino acids with a molecular weight of 50-kD [8]. It was originally thought to be expressed in retinal pigment epithelial cells as a neurotrophic factor, however, further in-depth research on PEDF has shown that it is also expressed in a variety of other cell types and tissues with various roles including the ability to inhibit angiogenesis, reduce inflammation, and promote cell survival under pathological conditions [9]. PEDF was considered to be a potential endogenous anti-inflammatory and antioxidant factor [10]. For example, it has been found that PEDF could inhibit the activation of NF-κB and the expression of a variety of pro-inflammatory genes, such as intercellular cell adhesion molecule 1, tumor necrosis factor alpha, and matrix metalloproteinases [11]. In addition, PEDF has also been shown to bind to its receptors to reduce retinal cell damage and apoptosis [12]. PEDF has been poorly studied in lung diseases, with some studies reaching relatively controversial conclusions. For example, recombinant PEDF was demonstrated to have a significant therapeutic effect on chronic ovalbumin-induced allergic mice by inhibiting eosinophilic airway inflammation and airway remodeling [13]. However, it may be involved in promoting the development of COPD by performing proinflammatory functions [14].

To date, there is still not enough research to investigate the biological role of PEDF in ALI. Therefore, the purposes of this study were to 1) explore whether PEDF can inhibit the occurrence and development of ALI and reduce epithelial cell damage, and 2) explore the relevant mechanism underlying PEDF's role in the process of ALI.

## Materials and methods

### Reagents and antibodies

Recombinant rat PEDF (GenBank accession number: NM_177927): Cusabio Biotech Co, Ltd (Wuhan, Hubei, China); The bacterial lipopolysaccharide (LPS) (L4391, O111:B4) and MPO colorimetric activity assay kits: Sigma (St. Louis, MO, USA). The LDH cytotoxicity assay kit and BCA protein concentration determination kit: Beyotime (Shanghai, China); Tissue and cell total protein extraction kits: Sangon Biotech (Shanghai, China). ELISA kits to measure TNF-α, IL-1β, and IL-6: Shanghai Renjie Biotechnology Co., Ltd. (Shanghai, China) [15]. ELISA kits to measure PEDF: Shanghai Yan Qi Biological Technology Co Ltd. The TUNEL kit, annexin V‑FITC/PI apoptosis detection kit, and DAPI staining solution: KeyGEN Biotech (Nanjing, Jiangsu, China). Anti-rabbit cleaved-caspase-3 (Catalog No. #9661), anti-rabbit p38 MAPK (Catalog No. #8690), anti-rabbit phospho-p38 MAPK (Catalog No. #4511), anti-rabbit NF-κB p65 (Catalog No. #8242), and anti-rabbit phospho-NF-κB p65 (Catalog No. #3033) antibodies: Cell Signaling Technology (Danvers, MA, USA). Anti-rabbit NLRP3 (Catalog No. ab263899), anti-rabbit PPAR-γ (Catalog No. ab178860) antibodies and PPAR-γ inhibitor (GW9662, Catalog No. ab141125): Abcam (Cambridge, MA, USA) [16]. Anti-rabbit RIP3 (Catalog No. 17563–1-AP) and anti-mouse β-tubulin (Catalog No. 66240–1-Ig) antibodies: Proteintech (Wuhan, Hubei, China).

### Animals

Sprague–Dawley (SD) male rats (250 ± 20 g, 8–10 weeks) were bought from the Experimental Animal Center of Xuzhou Medical University. The rats were maintained on a 12 h light–dark cycle with free access to food and water [16]. The rat care and experimental protocols were examined and verified by the Laboratory Animal Ethics Committee of Xuzhou Medical University.

### Rat ALI model and bronchoalveolar lavage fluid (BALF) collection

A total of 60 Sprague–Dawley rats were anesthetized with sodium pentobarbital (60 mg/kg) intraperitoneally. An ALI model was established by intratracheal instillation of LPS (2 mg/kg) using a 16G intravenous indwelling needle. The rats were then randomly divided into four groups as follows: (1) Normal group, no treatment, *n* = 10; (2) PEDF group, only injected with PEDF (5 mg/kg) 3 times via tail vein (every 6 h, a total dose of 15 mg/kg) [17], *n* = 10; (3) LPS group, where LPS was instilled into the trachea and rats were euthanized (intraperitoneal injection of sodium pentobarbital, 180 mg/kg) after 1d (*n* = 10), 2d (*n* = 10), or 4d (*n* = 10); (4) LPS + PEDF group (*n* = 10), where after 24 h of LPS stimulation, the tail vein was injected with PEDF (5 mg/kg) and this was performed three times at 6 h intervals, whereby rats were euthanized at the time point of 24 h after the first administration of PEDF (that is, 48 h after LPS stimulation). (5) LPS + PEDF + GW9662 group (*n* = 10), where after 24 h of LPS stimulation, GW9662 was injected intraperitoneally (2 mg/kg) while PEDF (5 mg/kg, three times at 6 h intervals, a total dose of 15 mg/kg) was injected [18], the euthanasia time of rats is the same as above (48 h after LPS stimulation). Half of the experimental animals were used to prepare BALF and lung tissue RNA. BALF was harvested from the lungs using a 16G intravenous indwelling needle with 5 mL of cold PBS (pH 7.4).

### Histologic assessment of lung tissue

Left lungs were immersed in 4% paraformaldehyde for 24 h, dehydrated, embedded in paraffin, and the resulting wax blocks were cut into 4-μm-thick sections. Then, sections were stained with hematoxylin and eosin (H&E). Pathological scores were calculated as described by Jianhua Fan et al. [19]. In brief, alveolar congestion, hemorrhage, degree of neutrophil infiltration, and thickness of alveolar wall/hyaline membrane were quantified into a 5-point score: 0 (minimal damage), 1 + (mild damage), 2 + (moderate damage), 3 + (severe damage), and 4 + (maximal damage). Pathological scores denoted as the sum of four aspects. Fifteen to 20 images were captured (400 × magnification), and the number of neutrophils was quantified by two blinded investigators [15].

### Lung wet/dry (W/D) ratio

The wet right lung tissue (no PBS perfused) from rats was weighed using a precision electronic balance. Next, the tissue was placed in a 60 °C incubator for 72 h to obtain dried lung tissue. The dry weight of the lung tissue was measured again to calculate the lung W/D ratio.

### Quantification of cytokines

The supernatant of BALF was collected by centrifugation (2000 g, 10 min). PEDF, TNF-α, IL-1β, and IL-6 levels were measured using quantitative ELISA kits according to the manufacturer’s protocols [15].

### Myeloperoxidase (MPO) assay

Lungs were perfused with PBS to remove all blood and then weighed for the MPO assay. MPO activity was detected according to the instructions of the MPO colorimetric activity assay kit [20].

### Reverse transcription‑quantitative polymerase chain reaction (RT‑qPCR) analysis

Total RNA was isolated from lung tissue with TRIzol reagent. Each 20 µl sample consists of 10 µl SYBR‑Green PCR Master mix (2X), 0.1 µM primers, 100 µg genomic DNA. The samples were amplified by qPCR using a Roche Light Cycler 480 following procedure: 95 °C 10 min, 45 cycles (95 °C 10 s, 60 °C 10 s, 72 °C 20 s), one cycle (95 °C 1 min, 65 °C 1 min, 97 °C continuous), 40 °C 30 s. Primers synthesized by Sangon Biotech (Shanghai, China), PEDF forward, 5'‑CAGAGTCTGTCATTCACCGGGC‑3'; reverse, 5'‑GTCAGCACAGCTTGGATAGTCTTC‑3'. β-actin forward, 5'‑ CTAAGGCCAACCGTGAAAAGA‑3' and reverse, 5'‑ CCAGAGGCATACAGGGACAAC‑3'. The level of PEDF mRNA were quantified through β-actin.

### Terminal deoxynucleotidyl transferase dUTP nick end labeling (TUNEL) staining for apoptosis

Lung tissue apoptosis in vivo was determined by TUNEL immunofluorescence staining, which was performed according to the manufacturer’s protocols. Nuclei were stained with DAPI at room temperature for 10 min to count the total number of cells. The rate of apoptotic cells was calculated as the ratio of the number of TUNEL‑positive cells to the total number of cells, which were counted in at least three different random fields of view [21].

### Cell culture and treatment

The rat lung epithelial type II RLE-6TN cell line was obtained from the Shanghai Zhongqiaoxinzhou Biotech, and the cells were cultured in DMEM culture medium containing 10% fetal bovine serum and incubated in a humidified atmosphere containing 5% CO_2_. The medium was replaced every 2–3 days, and cells were sub-cultured or subjected to experimental procedures at 80–90% confluence. LPS (2 μg/ml) was added for 4, 8, 12, 24 h to induce damage to RLE-6TN cells. RLE-6TN cells were stimulated with LPS for 24 h to assess the level of apoptosis, and the PEDF treatment group (LPS + PEDF group) was treated with recombinant rat PEDF (20 nM) after 2 h of LPS stimulation. Then, at the same LPS + PEDF concentration, 10 μM of GW9662 was added (LPS + PEDF + GW9662 group) at the point in time when the PEDF was added. The PEDF group was only treated with the same concentration of PEDF, and the normal group was not treated. Each experiment was repeated at least four times.

### Lactate dehydrogenase (LDH) cytotoxicity assay

Cell LDH release was detected by the LDH cytotoxicity assay kit according to the manufacturer's instructions.

### Western blotting analysis

Proteins were extracted from RLE-6TN cells using a cell total protein extraction kit, and protein concentrations were subsequently determined using a BCA protein concentration assay kit. Proteins were separated by SDS–PAGE and transferred onto nitrocellulose membranes. After blocking in 5% nonfat milk for 2 h, the membranes were incubated with primary antibodies against cleaved-caspase-3, rip3, NLRP3, p38 MAPK, phospho-p38 MAPK, NF-κB p65, phospho-NF-κB p65, PPAR-γ, or β-tubulin overnight at 4 °C. After washing, the membranes were incubated with either fluorescently labeled anti-mouse or anti-rabbit secondary antibodies at room temperature for 1–2 h, and the blot was then imaged using the Odyssey infrared imaging system (Li-Cor). Densitometric analysis of the bands was performed using ImageJ software. Protein levels were calculated from the ratio of corresponding protein/β-tubulin [15].

### Determination of apoptosis by flow cytometry

The annexin V‑FITC/PI apoptosis detection kit was used for conducting flow cytometry. Briefly, RLE-6TN cells (5 × 106 per group) were harvested, washed with PBS, resuspended in 500 µl binding buffer, treated with 5 µl annexin V‑FITC and 5 µl PI, and then incubated for 5–15 min at room temperature in the dark. Apoptosis rates were then calculated using a flow cytometer (BD Biosciences, NJ, USA).

### Immunofluorescence staining

The cell samples were fixed with 4% paraformaldehyde for 15–20 min, then blocked with a solution containing 5% bovine serum and 0.1% Triton X-100 for 1 h. Specimens were incubated with anti-mouse PPAR-γ antibody for 12 h at 4 °C and then incubated with the secondary antibody for 1 h at room temperature under dark conditions. The nucleus was stained with DAPI. After the last wash with PBS, the samples were observed under a fluorescence microscope (Olympus). Immunofluorescence intensity analysis of samples was performed using ImageJ software.

### Statistical analysis

All quantitative data were expressed as the mean ± standard deviation (SD). The student’s t-test was used for comparison between two groups, and the independent-samples t-test was used for comparisons between groups after evaluation by one-way ANOVA. Analyses were performed using the SPSS 25 software. Differences with *P* < 0.05 were considered statistically significant.

## Results

### The expression of PEDF decreased in LPS-induced rat ALI

**Fig. 1:**
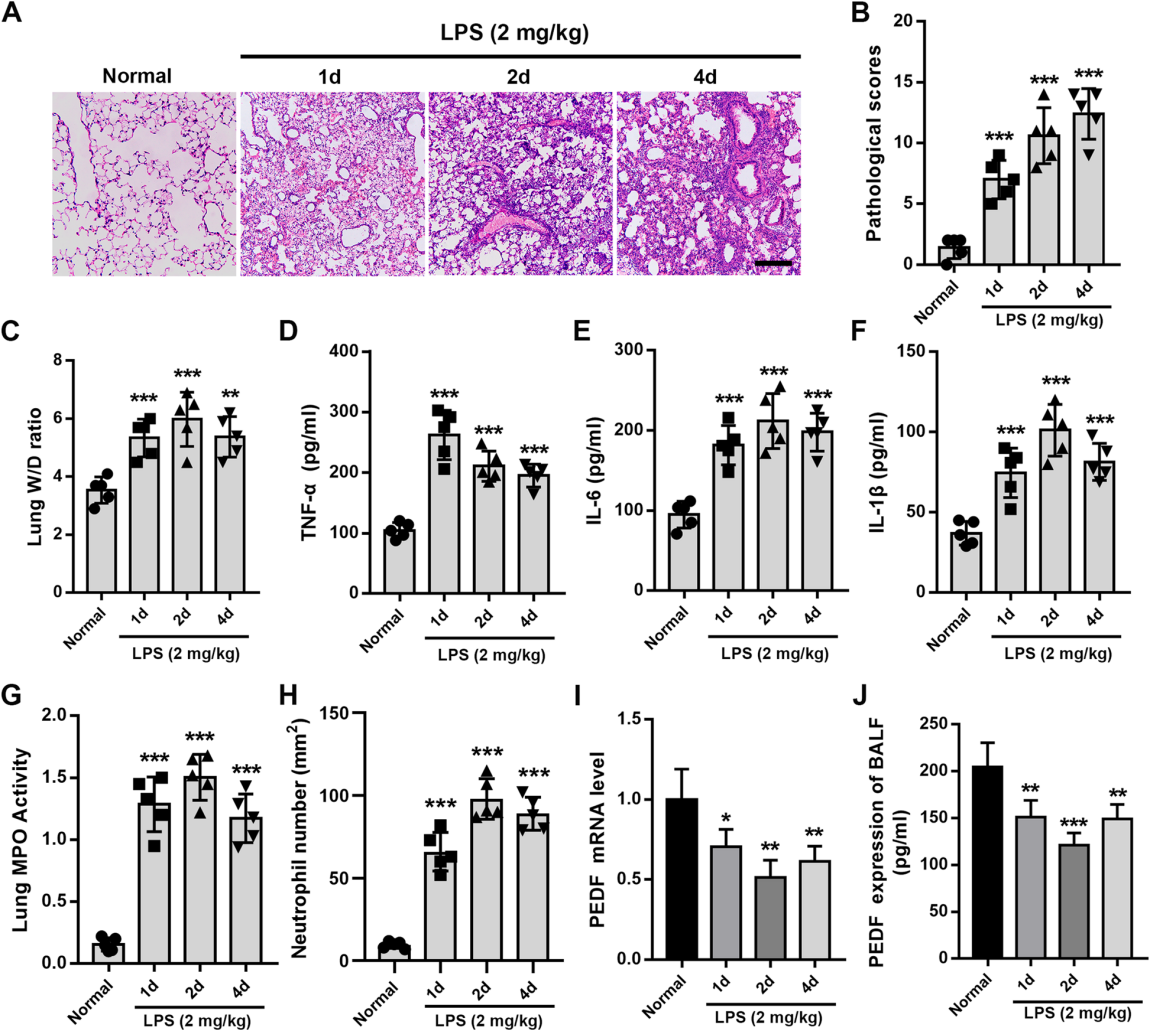
The expression of PEDF decreased during LPS-induced ALI in rats. **A** H&E-stained cross-section of the lung from LPS-exposed rats at the corresponding point in time. Scale bars: 200 μm. **B** The pathological scores of the Normal group and LPS (1d, 2d, 4d) group. *n* = 5. **C** Quantitative analysis of lung W/D ratio. Quantification of cytokines. **D** TNF-α, **E** IL-6, and **F** IL-1β expression levels. **G** Quantitative analysis of lung tissue MPO activity and **H** the number of neutrophils. **I** PEDF mRNA expression of lung tissue and **J** content of BALF. *n* = 5, Data are expressed as the mean ± SD, *P < 0.05; **P < 0.01; ***P < 0.001 vs. Normal group

The severity of LPS-induced rat ALI was investigated by histological analysis. We observed that the lung tissue of the rats was significantly damaged after 1d LPS attack, a large number of inflammatory cells had infiltrated the alveolar septum and alveolar cavity, and severe injury sustained for at least 4 days (Fig. 1A and B). The W/D ratio results also confirmed severe edema of the lung tissue (Fig. 1C). TNF-α, IL-6, and IL-1β are classic biomarkers representing inflammatory damage in lung tissue [22], their concentrations in the BALF were all significantly elevated (Fig. 1D–F). Consistent with this, the MPO activity and neutrophil counts, which represent the degree of neutrophil activation and aggregation [20], were also significantly increased (Fig. 1G and H). Next, we examined the expression of PEDF during rat ALI. The results showed that the expression level of PEDF was significantly lower than the normal level (Fig. 1I and J).

### PEDF reduced inflammation-induced damage and cell apoptosis in lung tissue

**Fig. 2:**
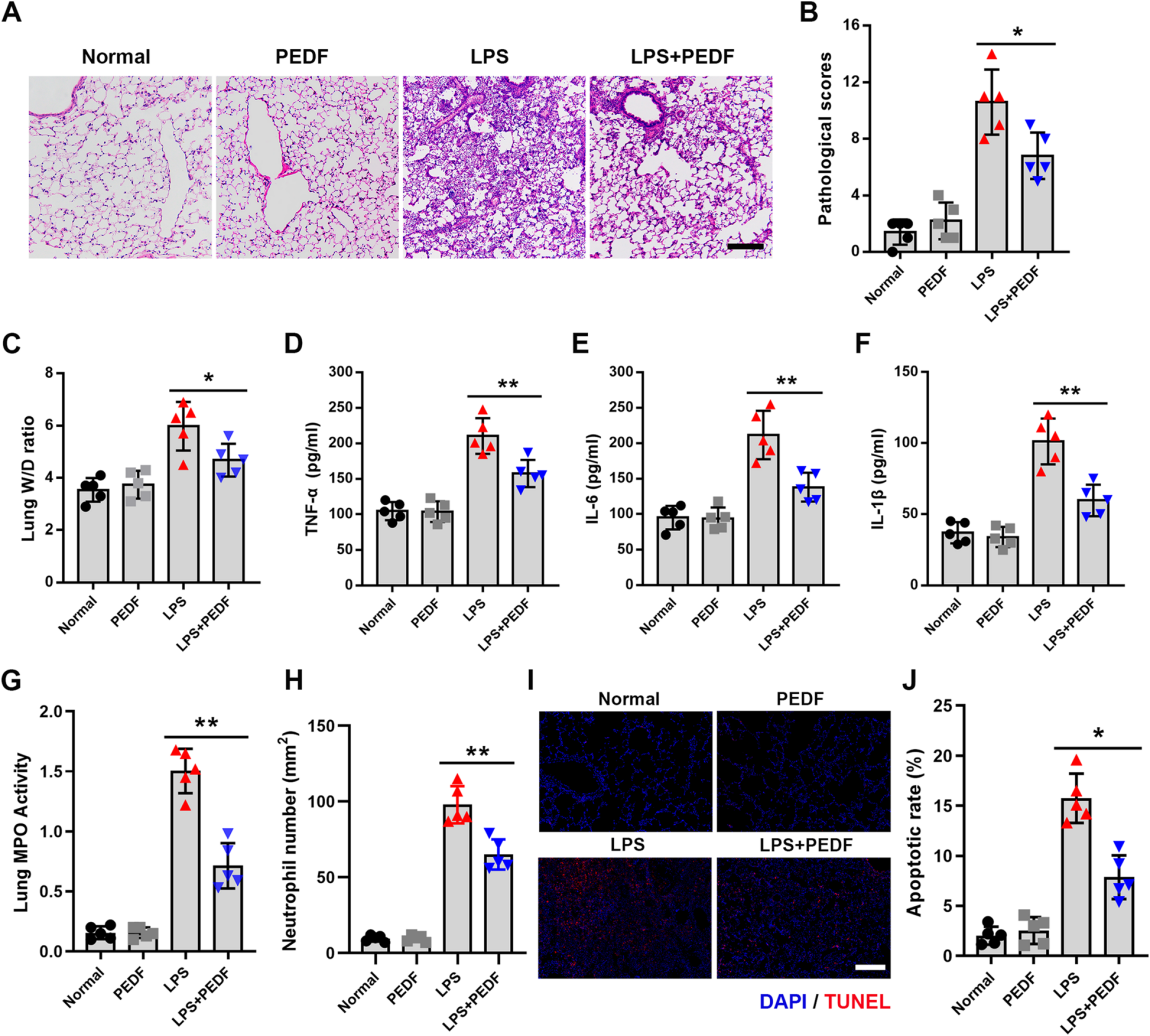
PEDF inhibited inflammatory injury and cell apoptosis in rat lung tissue. **A** H&E-stained cross-section of the lung from LPS-exposed rats with or without PEDF treatment. Scale bars: 200 μm. *n* = 5. **B** The pathological scores of each group. *n* = 5. **C** Quantitative analysis of lung W/D ratio. Quantification of cytokines. **D** TNF-α, **E** IL-6, and **F** IL-1β expression levels. **G** Quantitative analysis of lung tissue MPO activity and **H** the number of neutrophils. **I** Representative images of TUNEL staining of lung tissue. Scale bars: 200 μm. Images are representative of five animals. **J** Quantitative analysis of lung tissue apoptosis. *n* = 5, Data are expressed as the mean ± SD, *P < 0.05; **P < 0.01 vs. LPS group

According to the above results, to determine the therapeutic effect of PEDF in the early stages of ALI, we chose to inject PEDF 1d after LPS instillation, and then observed the treatment effect on the second day. The rats in the LPS-2d group were selected as the comparison objects. After receiving three injections of PEDF, the lung tissue structure was significantly improved, and the inflammatory cells in the alveolar cavity were also significantly reduced (Fig. 2A and B). The results of the lung tissue W/D ratio measurements also showed that PEDF was effective in reducing lung tissue edema (Fig. 2C). Next, we detected the concentration of inflammatory factors in BALF. After PEDF treatment, the expression levels of inflammatory factors decreased significantly (Fig. 2D–F), which was contingent on the results obtained for myeloperoxidase (MPO) activity and quantification of the number of neutrophils (Fig. 2G and H). These results indicated that PEDF had effective anti-inflammatory effects. Furthermore, we used TUNEL staining to investigate the apoptosis rate of lung tissues in each group. The results showed that LPS caused the extensive apoptosis of rat lung cells, and this phenomenon was effectively reversed by PEDF (Fig. 2I and J). Overall, these results supported that PEDF could reduce the lung tissue damage and apoptosis caused by LPS.

### PEDF reduced LPS-induced apoptosis of RLE-6TN cells

**Fig. 3:**
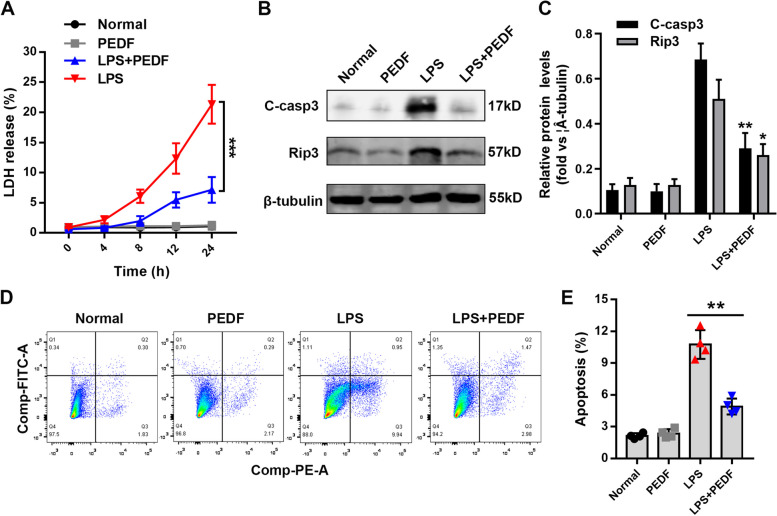
PEDF reduces LPS-induced apoptosis of RLE-6TN cells. **A** Quantitative analysis of LDH. **B** Western blotting to detect the effect of PEDF on the expression of c-casp3 (cleaved-caspase3) and rip3. **C** Quantification of the related protein expression. Data are expressed as the mean ± SD, *n* = 4, *P < 0.05 vs LPS group. **D** Flow cytometry to detect the effect of PEDF on cell apoptosis. **E** Quantification of the cell apoptosis. Data are expressed as the mean ± SD, *n* = 4, **P < 0.01 vs. LPS group

To further verify the effect of PEDF on lung epithelial cells, we cultured rat alveolar type II epithelial RLE-6TN cells in vitro and added LPS to establish a lung epithelial cell injury model [23]. We observed that with time, LPS significantly induced the release of LDH from RLE-6TN cells, while PEDF inhibited this process (Fig. 3A). Quantitative detection of cleaved-caspase3 and rip3 also confirmed that PEDF could effectively reduce LPS-mediated RLE-6TN cell damage and apoptosis (Fig. 3B and C). Finally, we used flow cytometry to quantify the rate of apoptosis of RLE-6TN cells. After 24 h of LPS treatment, the apoptotic rate increased significantly compared to that of the normal group, and after PEDF intervention, the apoptosis rate decreased significantly (Fig. 3D and E). This further confirmed the protective effect of PEDF on epithelial cells.

### PEDF inhibited LPS-induced inflammatory damage in RLE-6TN cells

**Fig. 4:**
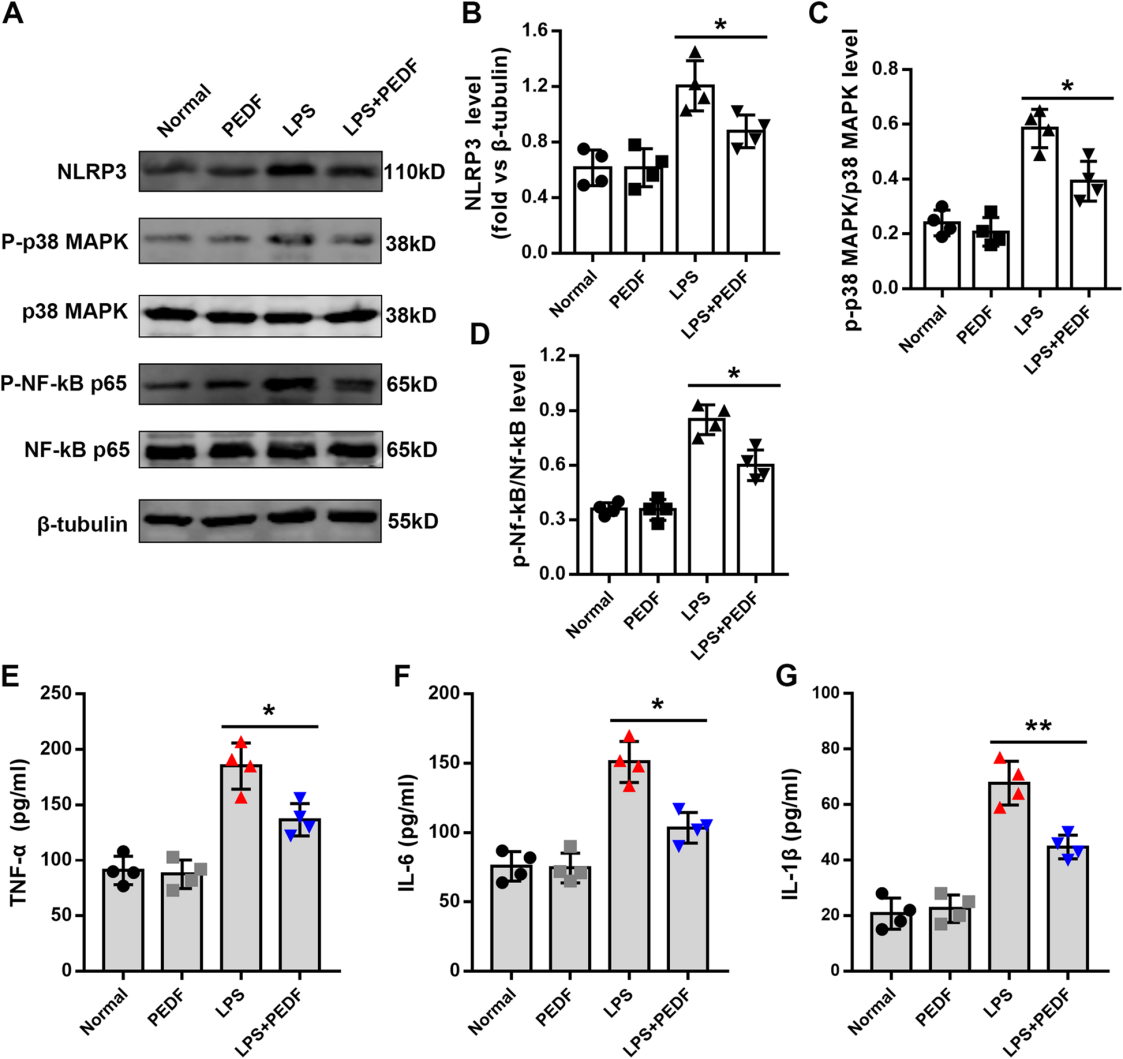
PEDF reduces LPS-induced inflammatory damage and cytokines. **A** Immunoblot analysis of NLRP3, p38 MAPK, phospho-p38 MAPK, NF-κB p65 and phospho-NF-κB p65. **B**, **C** and **D** Quantification of the related protein expression. *n* = 4, Data are expressed as the mean ± SD, *P < 0.05. Quantification of cytokine concentrations in the cell supernatant. **E** TNF-α, **F** IL-6, and **G** IL-1β expression levels. Data are expressed as the mean ± SD, *n* = 4, *P < 0.05; **P < 0.01 vs. LPS group

After preliminary results confirming the protective effect of PEDF on epithelial cells, we next explored the relevant effects of PEDF on inflammatory damage. Bacterial lipopolysaccharide activates inflammatory pathways such as NLRP3, p38 MAPK, and NF-κB through Toll-like receptors, thereby ultimately mediating inflammatory damage and the apoptosis of cells [24]. We determined that LPS promoted the phosphorylation levels of both NF-κB p65 and p38 MAPK, as well as the expression level of NLRP3. Pigment epithelium-derived factor partially reversed the above-mentioned protein changes (Fig. 4A–D). The levels of inflammatory factors (TNF-α, IL-6, and IL-1β) that were detected from the cell supernatant were also significantly reduced by PEDF treatment (Fig. 4E–G). These findings collectively revealed that PEDF mediated the protective effect of epithelial cells by inhibiting inflammatory damage.

### PEDF inhibited LPS-induced inflammatory damage by upregulating PPAR-γ

**Fig. 5:**
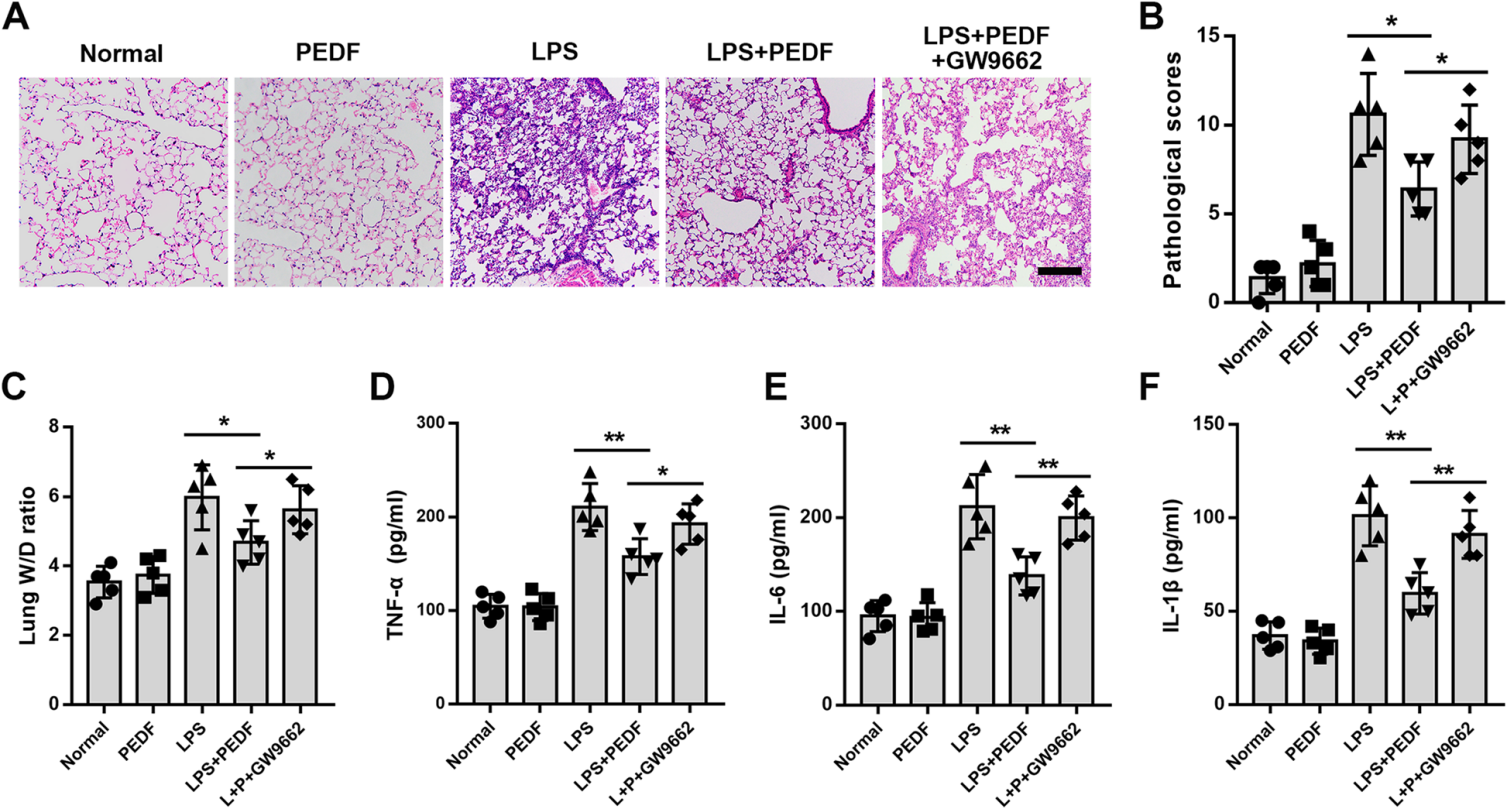
PPAR-γ inhibitor (GW9662) reverses the protective effect of PEDF in rat ALI model. **A** H&E-stained cross-section of the lung tissue of each group. Scale bars: 200 μm. *n* = 5. **B** The pathological scores of each group. *n* = 5. **C** Quantitative analysis of lung W/D ratio. Quantification of cytokines. **D** TNF-α, **E** IL-6, and **F** IL-1β expression levels. *n* = 5, Data are expressed as the mean ± SD, *P < 0.05; **P < 0.01 vs. indicated group

**Fig. 6:**
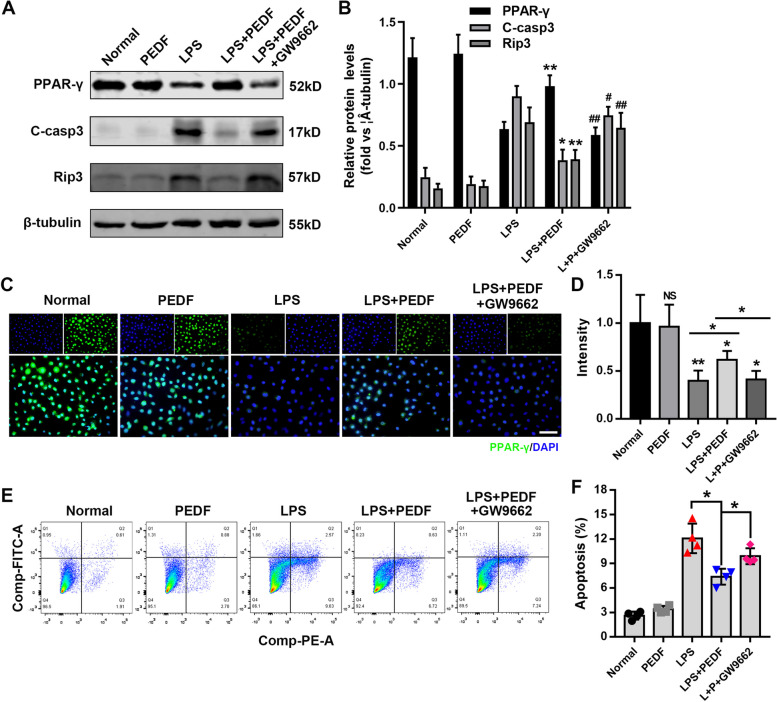
PEDF reduces LPS-induced apoptosis of RLE-6TN cells by upregulating PPAR-γ. **A** Western blotting to detect the expression of cleaved-caspase3 and rip3 to investigate the effect of the PPAR-γ inhibitor on PEDF treatment. **B** Quantification of the related protein expression. Data are expressed as the mean ± SD, *n* = 4. **C** Representative images of PPAR-γ immunofluorescence. Scale bars: 50 μm. **D** Quantification of the PPAR-γ fluorescence density. **E** Flow cytometry to detect the effect of the PPAR-γ inhibitor on PEDF treatment. **F** Quantification of the cell apoptosis. Data are expressed as the mean ± SD, *n* = 4, *P < 0.05, **P < 0.01 vs. LPS group or indicated group; #*P* < 0.05, ##*P* < 0.01 vs LPS + PEDF group

**Fig. 7:**
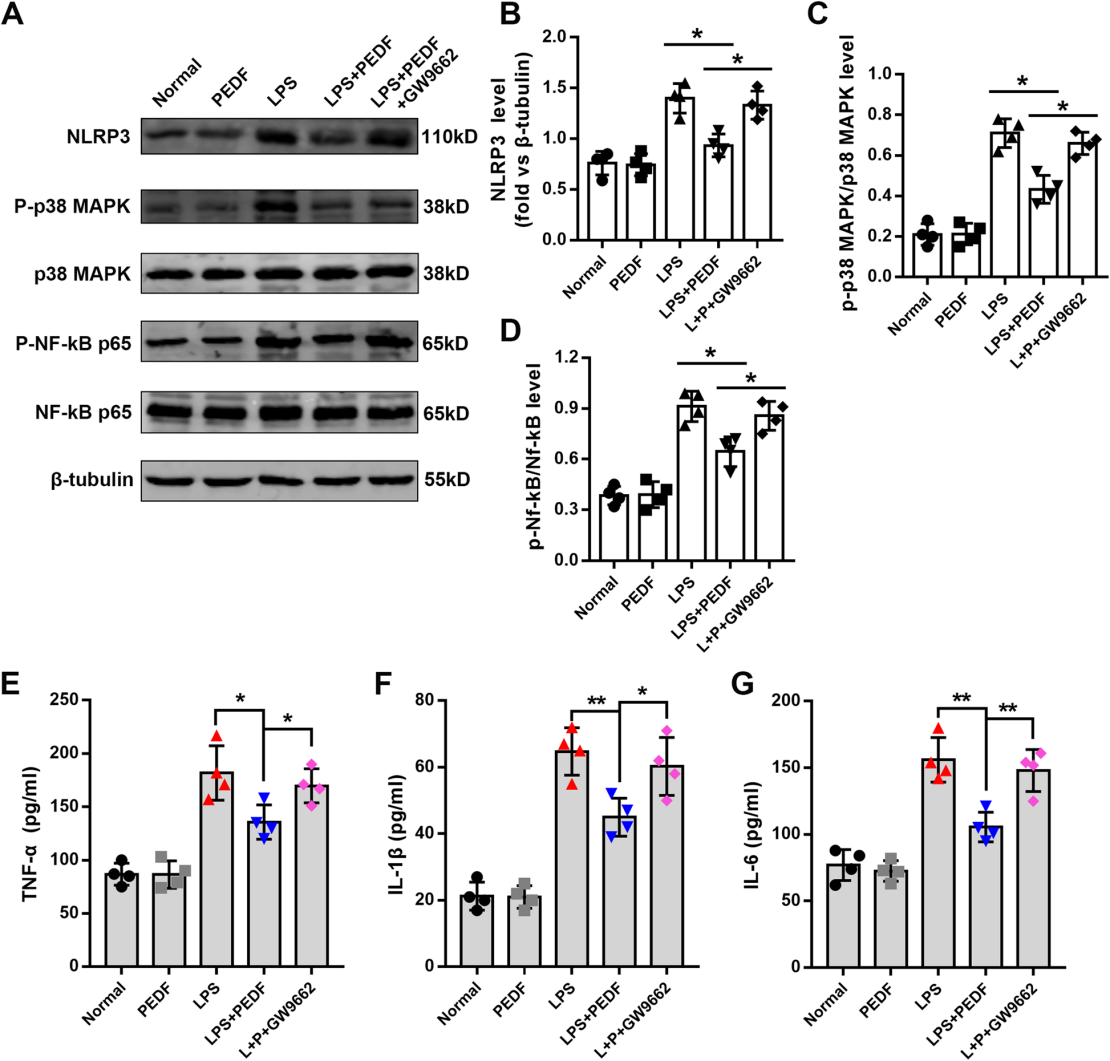
PEDF reduced LPS-induced inflammatory in RLE-6TN cells by upregulating PPAR-γ. **A** Immunoblot analysis of NLRP3, p38 MAPK, phospho-p38 MAPK, NF-κB p65, phospho-NF-κB p65. **B**, **C** and **D** Quantification of the related protein expression. Quantification of TNF-α (**E**), IL-6 (**F**), and IL-1β (**G**) concentrations in the cell supernatant. Data are expressed as the mean ± SD, *n* = 4, *P < 0.05; **P < 0.01 vs. indicated group

After demonstrating the protective effect of PEDF on ALI, we investigated the molecular mechanism by which PEDF could inhibit epithelial cell apoptosis. Peroxisome proliferator-activated receptor γ (PPAR-γ) is a transcription factor that can heterodimerize with retinoid X receptors to activate genes involved in lipid homeostasis [25]. Studies have demonstrated that PPAR-γ expression is reduced in lung tissue during ALI, and that PPAR-γ activators or overexpression of PPAR-γ could inhibit lung injury progression [26, 27]. Previous studies have shown that PEDF could significantly upregulate PPAR-γ and that this was an important mechanism for protecting the survival of ischemic cardiomyocytes [28, 29]. Whether PEDF upregulates the expression of epithelial cell PPAR-γ and affects the process of inflammatory injury remains to be determined. Consistent with our expectations, in rat models of lung injury induced by LPS, PPAR-γ inhibitor (GW9662) reversed the protective effect of PEDF and aggravated lung injury (Fig. 5A–F). In addition, following LPS stimulation, the PPAR-γ expression of RLE-6TN cells was reduced significantly, and recombinant rat PEDF reversed this change (Fig. 6A–D). The apoptosis rate of RLE-6TN cells treated with the inhibitor GW9662 (PPAR-γ ligand binding domain antagonist) increased (Fig. 6A, B, E and F). Additionally, GW9662 reversed the anti-inflammatory effect of PEDF (Fig. 7A–G). In summary, our findings indicate that PEDF promoted lung epithelial cell survival by upregulating PPAR-γ.

## Discussion

In this study, PEDF was shown to reduce lung tissue damage in a rat LPS-induced model, reduce inflammatory factor expression, and reduce lung epithelial cell apoptosis. Furthermore, the mechanism of its biological function was confirmed: PEDF inhibits NLRP3 activation and promotes the survival of lung epithelial cells by enhancing the expression of PPAR-γ, thereby reducing LPS-induced ALI.

There are many risk factors for ALI, the animal model of ALI is an important means by which to study the pathogenesis of ALI and explore its treatment options. Although no animal model can fully replicate all the characteristics of human ALI, the animal model of LPS-induced ALI can replicate the pathogenesis and prognosis of ALI well [30]. Bacterial lipopolysaccharide is the main component of endotoxin. After entering the body, it binds to Toll-like receptors on the cell surface and is then activated by the MyD88-dependent signaling pathway and cell signal transmission. It then activates inflammatory signal regulatory proteins such as NLRP3 and NF-κB to produce a cascade of inflammatory effects, ultimately leading to extensive damage to lung tissues and cells [31, 32]. Inhibition of inflammatory signaling pathways such as NLRP3 and NF-κB can greatly reduce the severity of ALI-related inflammation [33, 34]. The stimulation of LPS has been shown to lead to inflammatory lung injury dominated by alveoli, abnormal changes in lung tissue, pathological characteristics, and a significant increase in pro-inflammatory factors [35]. Additionally, the activation of neutrophils has been shown to promote the expression of lung cytotoxic products, such as the overproduction of both oxygen free radicals and granzymes in lung tissue [36].

Pigment epithelium-derived factor was originally described as a neurotrophic factor but has been further recognized as one of the most effective endogenous antiangiogenic factors [9]. The antitumor and anti-inflammatory effects of PEDF have also gradually been confirmed [37]. Current research focuses on evaluating the therapeutic potential of PEDF for tumors, cardiovascular diseases, and other pathological conditions. However, to our knowledge, there is still a lack of sufficient research to explore the role of PEDF in respiratory diseases. We found that after subcutaneous injection of recombinant rat PEDF, the concentration of inflammatory factors detected in BALF decreased significantly. Furthermore, the MPO value; which reflects the degree of activation of neutrophils [20]; also decreased significantly. More importantly, we observed a widespread decrease in the lung cell apoptosis rate, which further confirms the protective effect of PEDF on rat lung tissue. To clarify the specific effect of PEDF on lung epithelial cells in in vitro experiments, we administered LPS to induce the apoptosis of RLE-6TN cells, and this process was demonstrably inhibited by PEDF. Bacterial lipopolysaccharide significantly activated the expression of NLRP3 and the phosphorylation of NF-κB p65, which is an important signaling pathway that triggers inflammation. After adding exogenous recombinant rat PEDF, the expression of the above-mentioned protein decreased significantly. Flow cytometry results also suggested that PEDF can promote the survival of epithelial cells under injury conditions. Many studies have shown that PPAR-γ regulates inflammation and cell survival, and upregulation of PPAR-γ has also been shown to be anti-inflammatory, antioxidant, and anti-apoptotic [26, 27]. It is worth noting that the protective effect of PEDF was reversed to some extent by PPAR-γ inhibitors, indicating that PEDF protected epithelial cells at least partly by regulating PPAR-γ.

Our study has some limitations. PEDF could bind to multiple cell surface receptors [12], and its target on epithelial cells was unclear. Damage to the endothelial barrier is also a typical feature of ALI, and the angiogenesis is also an important cause of inflammation [20]. The effect of PEDF on the vascular barrier during ALI is unknown. PEDF is one of the strongest inhibitors of angiogenesis, interestingly, it tends to inhibit immature new blood vessels while protecting mature blood vessels [16, 38]. In the physiological environment of pathological injury, PEDF can be used as a multifunctional regulator to exert a multifaceted protective effect [39]. For other types of pulmonary cell damage and the improvement of lung injury environment, the specific mechanisms of PEDF biological function still need to be further explored.

## Conclusion

Overall, our results suggest that PEDF could be used as an anti-inflammatory factor following ALI. The application of PEDF during ALI is a feasible strategy to reduce epithelial cell apoptosis and improve lung tissue damage. Our findings contribute to novel applications of PEDF in respiratory diseases and provide a promising and prospective method for improving ALI management and prognosis strategies.

### Supplementary Information

**Additional file 1.**

## Data Availability

All data from this study are available in this published article.
