# Supplementary material for: PEDF inhibits LPS-induced acute lung injury in rats and promotes lung epithelial cell survival by upregulating PPAR-γ
Source: BMC Pulm Med. 2023 Sep 23;23:359. doi: 10.1186/s12890-023-02666-3 (PMC10517507; doi:10.1186/s12890-023-02666-3)

Cleaved-caspase3 for Fig3B

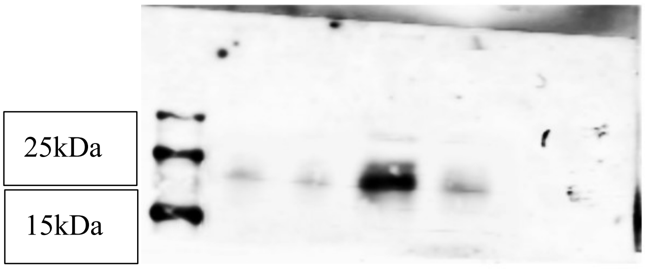

Rip3 for Fig3B

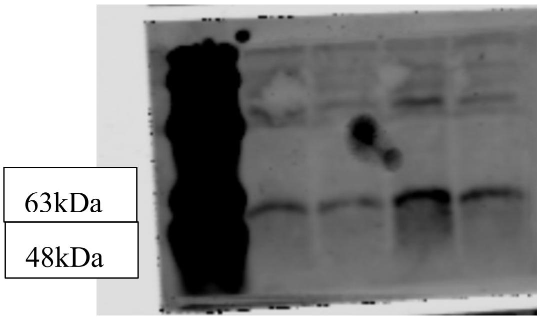

β-tubulin for Fig3B

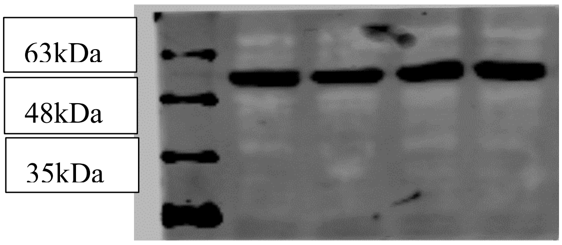

NLRP3 for Fig4A

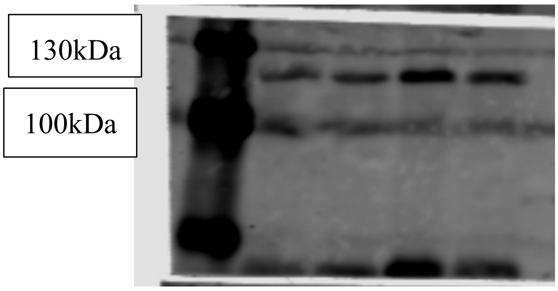

P-p38 MAPK for Fig4A

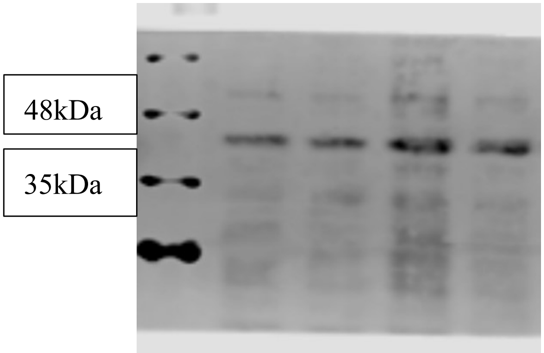

p38 MAPK for Fig4A

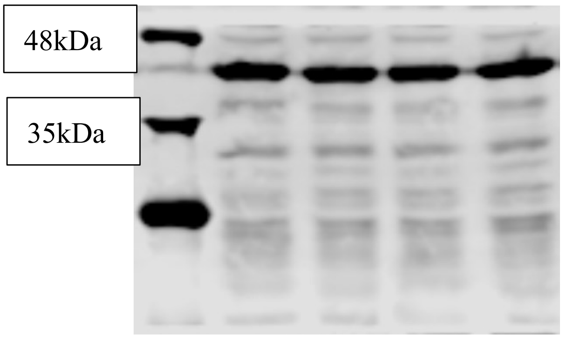

P-NF-kB p65 for Fig4A

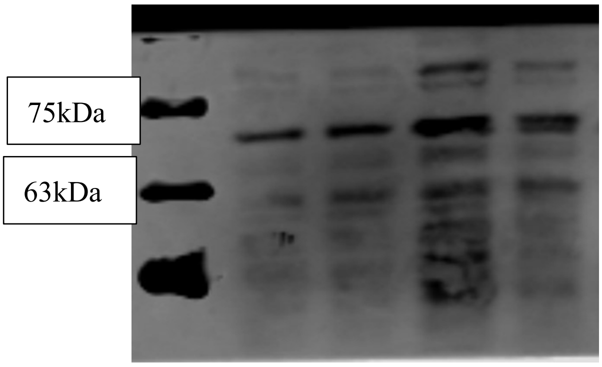

NF-kB p65 for Fig4A

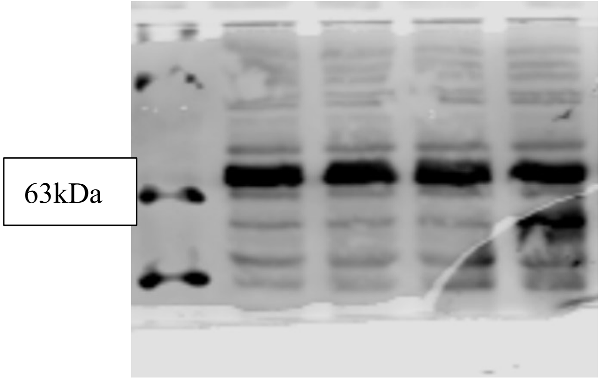

β-tubulin for Fig4A

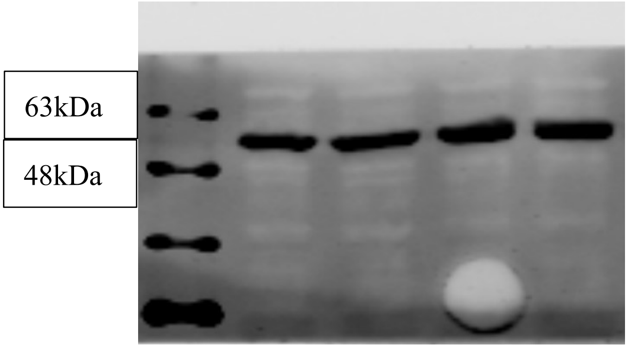

PPAR-γ for Fig6A

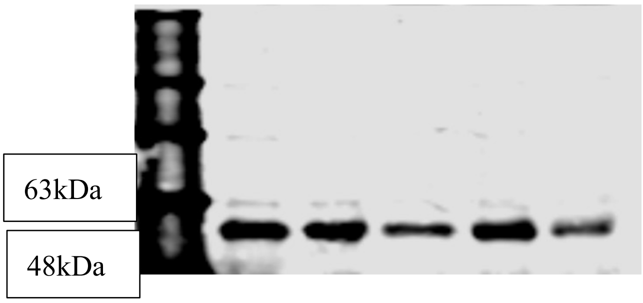

Cleaved-caspase3 for Fig6A

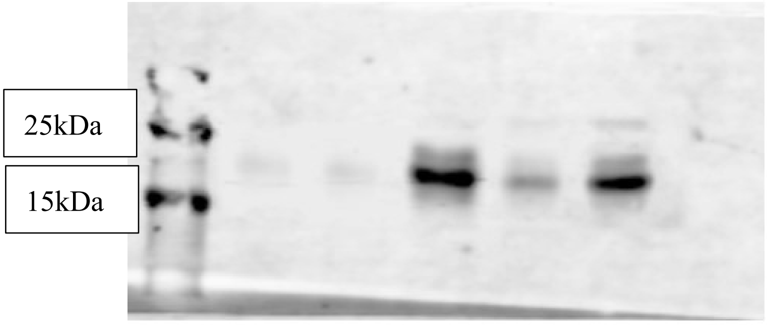

Rip3 for Fig6A

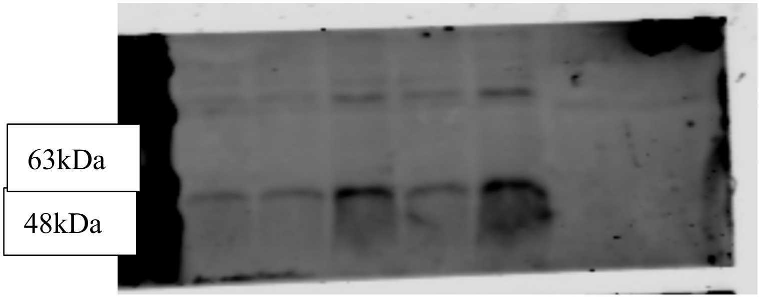

β-tubulin for Fig7A

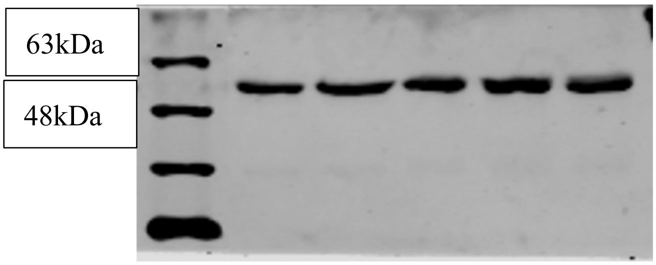

NLRP3 for Fig7A

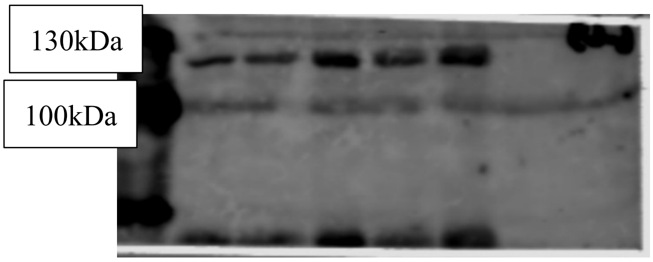

P-p38 MAPK for Fig7A

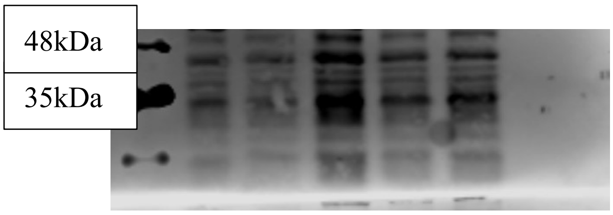

p38 MAPK for Fig7A

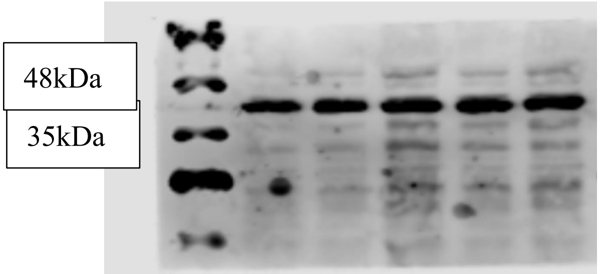

P-NF-kB p65 for Fig7A

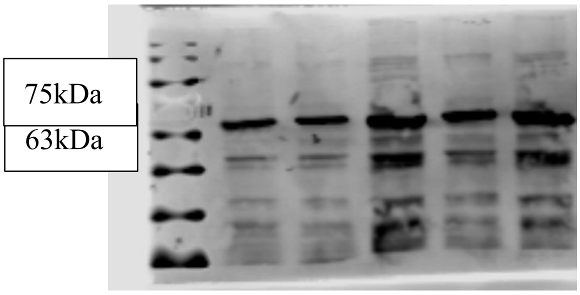

NF-kB p65 for Fig7A

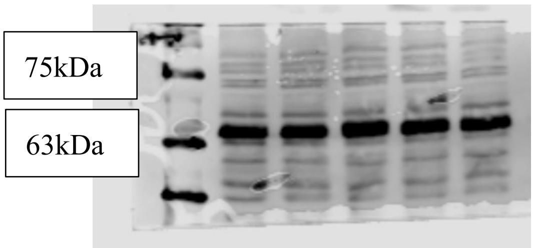

β-tubulin for Fig7A

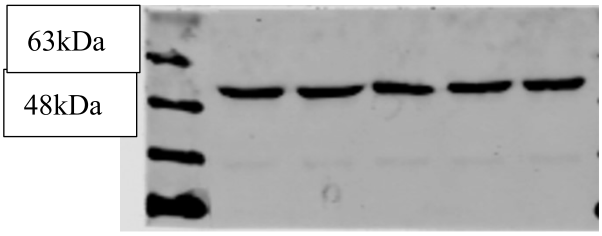

Supplement: Supplementary file 1 — Additional file 1. [file 12890_2023_2666_MOESM1_ESM.pdf]
